# The Strength of the Movement-related Somatosensory Cortical Oscillations Differ between Adolescents and Adults

**DOI:** 10.1038/s41598-019-55004-1

**Published:** 2019-12-06

**Authors:** James E. Gehringer, David J. Arpin, Jacy R. VerMaas, Michael P. Trevarrow, Tony W. Wilson, Max J. Kurz

**Affiliations:** 1 Center for Magnetoencephalography, University of Nebraska Medical Center, Omaha, NE United States; 2 Department Physical Therapy, Munroe Meyer Institute, University of Nebraska Medical Center, Omaha, NE United States; 3 Department of Neurological Sciences, UNMC, Omaha, Nebraska United States

**Keywords:** Cortex, Sensorimotor processing

## Abstract

Adolescents demonstrate increasing mastery of motor actions with age. One prevailing hypothesis is that maturation of the somatosensory system during adolescence contributes to the improved motor control. However, limited efforts have been made to determine if somatosensory cortical processing is different in adolescents during movement. In this study, we used magnetoencephalographic brain imaging to begin addressing this knowledge gap by applying an electrical stimulation to the tibial nerve as adolescents (Age = 14.8 ± 2.5 yrs.) and adults (Age = 36.8 ± 5.0 yrs.) produced an isometric ankle plantarflexion force, or sat with no motor activity. Our results showed strong somatosensory cortical oscillations for both conditions in the alpha-beta (8–30 Hz) and gamma (38–80 Hz) ranges that occurred immediately after the stimulation (0–125 ms), and a beta (18–26 Hz) oscillatory response shortly thereafter (300–400 ms). Compared with the passive condition, all of these frequency specific cortical oscillations were attenuated while producing the ankle force. The attenuation of the alpha-beta response was greater in adolescents, while the adults had a greater attenuation of the beta response. These results imply that altered attenuation of the somatosensory cortical oscillations might be central to the under-developed somatosensory processing and motor performance characteristics in adolescents.

## Introduction

Adolescents demonstrate greater mastery of single joint movements, including drawing, aiming, reaching and grasping objects as they become older^1–7^. Although this is a common finding, there is no consensus on why motor control improves during this developmental stage. One prevailing hypothesis is that maturation of the somatosensory system during adolescence might contribute to improved motor control^8–12^. Essentially, adolescents may have a diminished ability to detect errors in their selected motor actions because their interpretation of the sensory feedback is less precise and delayed^8,9,13–16^. Alternatively, other investigations have hypothesized that the motor control differences may not be related to the quality of the sensory feedback, but rather adolescents are less experienced at properly weighting all of the available sensory feedback during a movement (*i.e*.., muscle spindle, joint position, visual tracking)^10–12^. While both of these hypotheses are plausible, limited efforts have been made to determine if there is a connection between the somatosensory cortical processing and the motor actions seen in adolescents.

Principally, insight into movement-related somatosensory attenuation (i.e., gating) has come from peripheral nerve stimulation investigations focusing on event related potentials (ERP)^17–20^. Overall these studies have shown that the amplitude of the evoked somatosensory cortical activity is attenuated during movement. Although these outcomes have been essential for providing insight into somatosensory gating during a motor action and sensorimotor integration, cortical oscillations are likely to play a role in this computational processing, and this field remains mostly unexplored. Investigating the cortical oscillations may advance our understanding of the neural dynamics that are not directly phase-locked to the stimulation of the periphery. It is well established that stimulating the peripheral receptors of the foot during passive sitting generates an immediate and transient synchronization (e.g., increase in power) of the oscillatory activity in the somatosensory cortices across the 10–75 Hz frequency bands^21–24^. These neural synchronizations are generally followed by a desynchronization (e.g., decrease in power) stretching across the alpha (8–16 Hz) and beta (18–26 Hz) frequency bands that extends from about 150 to 400 ms. It has also been shown that neural synchronizations within the more limited theta-beta frequency range (6–24 Hz) are sustained while performing a haptic task, while the other frequency bands that were seen in the no movement condition are completely gated^25^. Although our knowledgebase on how changes in the somatosensory cortical oscillations reflect differences in sensory processing is rapidly expanding, whether these cortical oscillations are different between adolescents and adults during movement remains unknown.

In the present study, magnetoencephalographic (MEG) brain imaging was used to begin addressing this knowledge gap by stimulating the tibial nerve with an electrical pulse as adolescents and adults generated an isometric ankle plantarflexion force, or sat quietly with no motor activity (e.g., passive condition). Our key hypotheses were: (1) that for both groups the strength of the somatosensory cortical oscillations would be altered while producing the isometric force relative to the passive condition, and (2) while producing the isometric force, there will be a significant difference in the attenuation magnitude of the somatosensory cortical oscillations between the adolescents and adults.

## Results

### Sensor-level results

**Figure 1:**
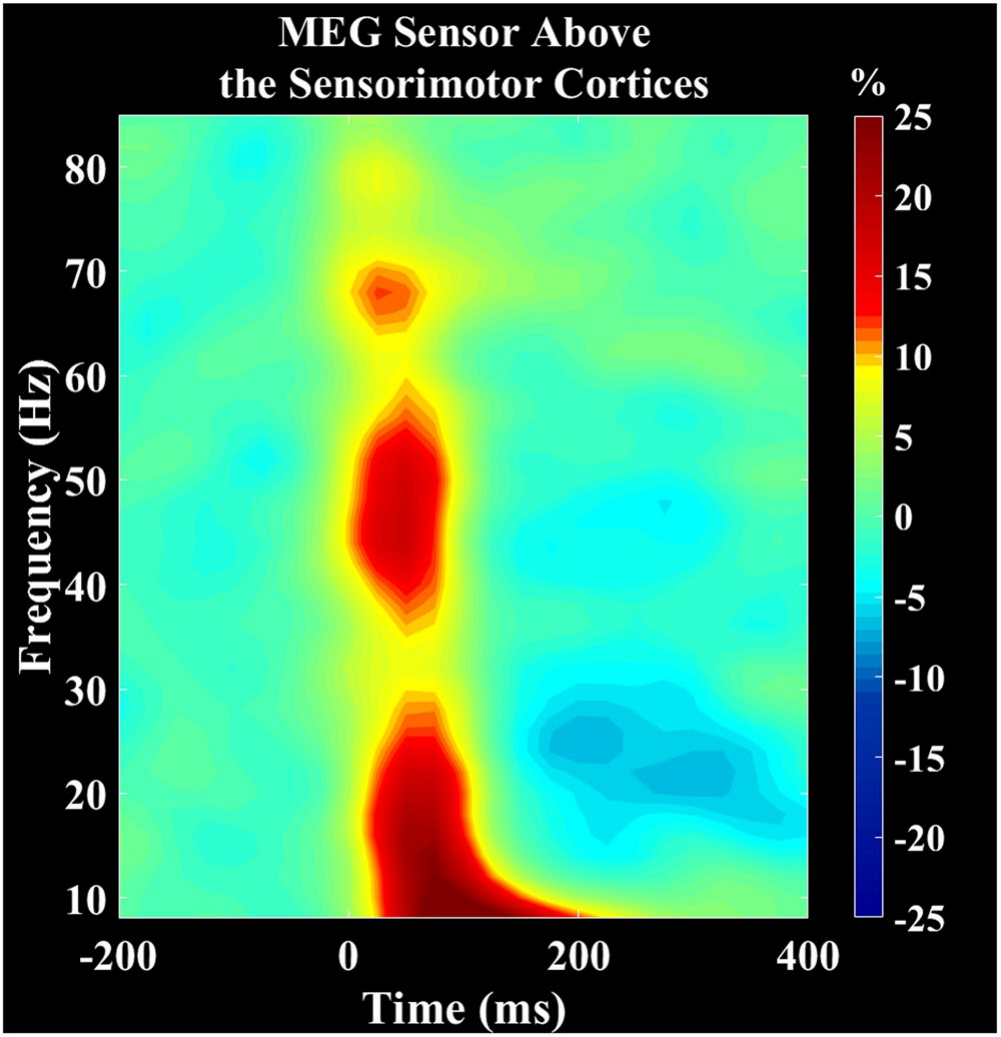
Grand averaged time-frequency spectrograms collapsed across group and conditions. Frequency (Hz) is shown on the y-axis and time (s) is denoted on the x-axis, with 0 ms defined as stimulation onset. The event-related spectral changes after the stimulation are expressed as percent difference from baseline (−200 to 0 ms). The MEG gradiometer with the greatest response amplitude was located near the medial sensorimotor cortices, contralateral to the ankle used during the task. There was a strong event related synchronization (ERS) in the alpha-beta (8–30 Hz, 0 to 125 ms) and gamma (38–80 Hz, 0 to 100 ms) bands for both the passive and active conditions. Additionally, there was an event related desynchronization (ERD) in the beta (18–26 Hz, 300 to 400 ms). The color scale bar is shown on the right.

The sensor-level MEG data were collapsed across the respective conditions (active and passive) and age groups and examined using the two-stage permutation based approach (Fig. 1). This revealed significant alpha-beta (8–30 Hz) and gamma (38–80 Hz) event related synchronizations (ERS) in a cluster of fronto-parietal sensors that began immediately after the stimulation and were sustained for 125 ms and 100 ms, respectively (*P* < 0.0001, corrected). In addition, a significant beta (18–26 Hz) event related desynchronization (ERD) was observed during the latter 300–400 ms time window (*P* < 0.0001, corrected).

### Gamma oscillations

**Figure 2:**
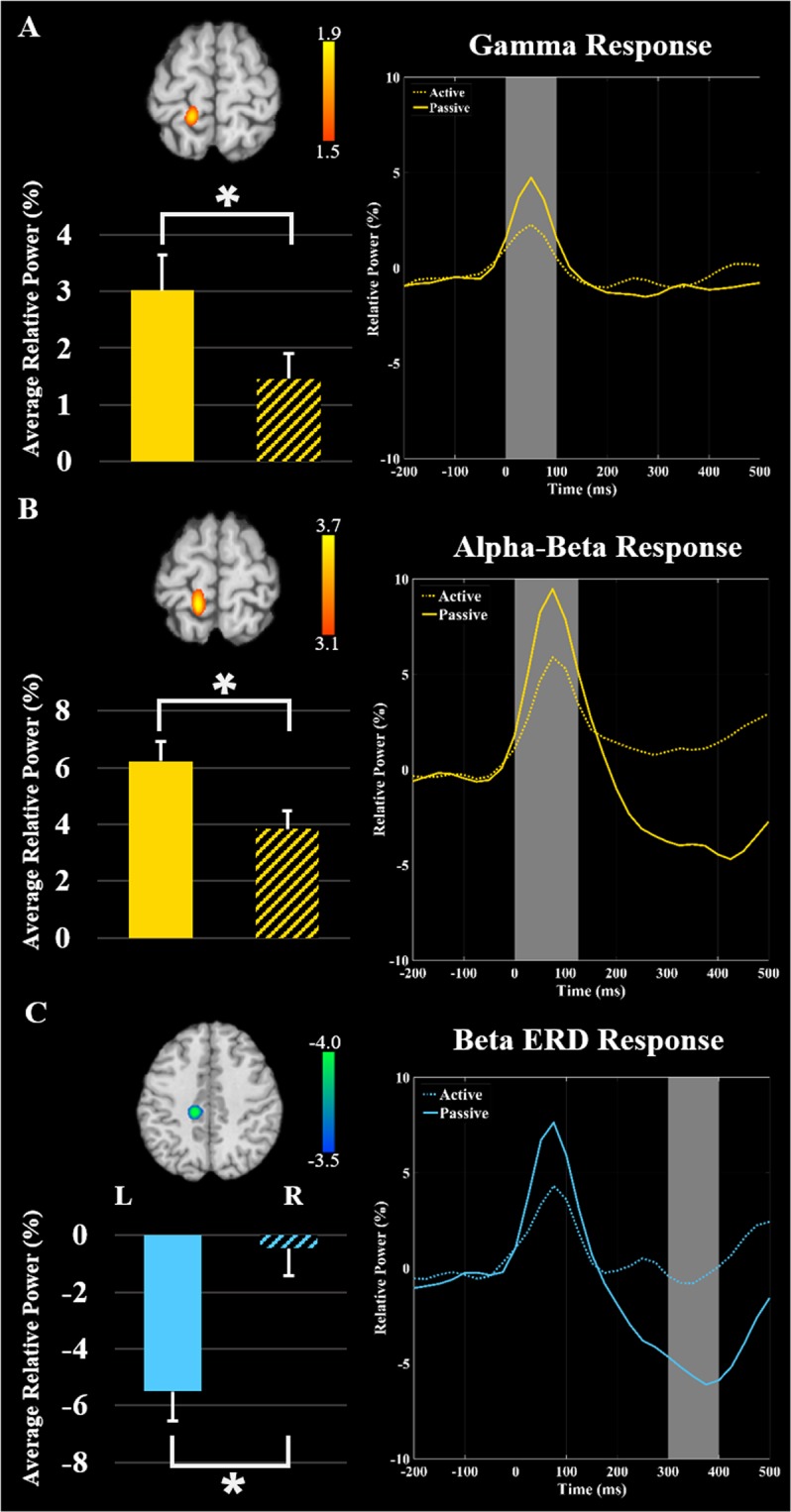
Grand averaged beamformer images collapsed across active and passive conditions and all participants for (**A**) gamma activity (38–80 Hz) from 0 to 100 ms, (**B**) alpha-beta activity (8–30 Hz) from 0 to 125 ms, and (**C**) beta oscillations (18–26 Hz) from 300 to 400 ms revealed strong clusters in the contralateral somatosensory cortex. Scale bars represent pseudo-t values. The neural time series shown to the right were extracted from the peak voxel in respective beamformer images. The solid line represents the neural time course during the passive condition, while the dash line repesents the active condition. The bar graphs represent the average relative power from 0 to 100 ms for gamma activity (38–80 Hz), 0 to 125 ms for alpha-beta activity (8–30 Hz), and 300 to 400 ms for beta oscillations (18–26 Hz). The windows have been shaded gray across the three panels. Significant power differences are denoted by the asterisk (P ≤ 0.05). As shown, the strength of somatosensory cortical activity was significantly weaker (*e.g*., gated) when participants generated the isometric ankle plantarflexion force (i.e., during the active condition).

To image the gamma (38–80 Hz) ERS, a beamformer was applied to the 0 to 100 ms time window using data collapsed across the respective conditions and a baseline period of −125 to −25 ms. The resulting images were then averaged across groups and this revealed that the gamma ERS was generated by the leg region of the contralateral somatosensory cortex (Fig. 2A). The local maximum seen in this cortical area was subsequently used to extract virtual sensor time courses for each condition per participant, and the average activity across the 0 to 100 ms time window was subsequently calculated. There was a significant difference in the power of the somatosensory response between conditions, indicating that the strength of the gamma ERS was weaker during the active condition (*P* = 0.014, Fig. 2A). However, there was no difference in the amount of attenuation between the groups (*P* = 0.67). Thus, the attenuation of the gamma ERS in the active condition was similar between the adolescents and adults.

### Alpha-beta event-related synchronization

**Figure 3:**
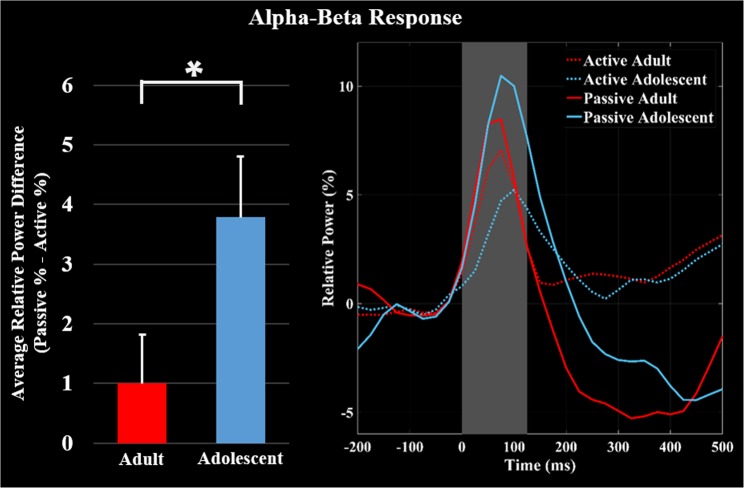
Average of the neural time courses per condition and group. Time series were extracted from the peak voxel in the alpha-beta grand averaged beamformer images as above, except now they are separated for the adolescents (blue) and adults (red). The solid lines represent the neural time course for the passive condition, while the dashed lines represent the neural time course for the active condition. The bar graph shows the amount of attenuation (Passive – Active) of the average relative power of alpha-beta event related synchronization (ERS) during the 0–125 ms time window. Significant differences in the magnitude of attenuation are denoted by the asterisk (P ≤ 0.05). As shown, the adolescents had greater attenuation (*e.g*., gating) of the alpha-beta ERS during the isometric ankle plantarflexion task compared to the adults.

As with the gamma response, the alpha-beta (8–30 Hz) ERS was imaged using data collapsed across the respective conditions (i.e., active and passive trials). The imaging window was 0 to 125 ms and a baseline period of −150 to −25 ms was used. The results indicated that the alpha-beta ERS also originated in the leg region of the contralateral somatosensory cortex (Fig. 2B). The maxima in this cortical area was subsequently used to extract the virtual sensor time series separately for the active and passive condition for each participant to show the source space power, and the average activity across the 0 to 125 ms time window was calculated. Statistical analyses indicated a significant difference in the power of the alpha-beta ERS between conditions (*P* = 0.016, Fig. 2B), which revealed that the response was significantly weaker during the active condition in the somatosensory cortices. Additionally, the attenuation of the alpha-beta ERS during the active condition was greater in the adolescents (*P* = 0.045, Fig. 3).

### Beta event-related desynchronization

**Figure 4:**
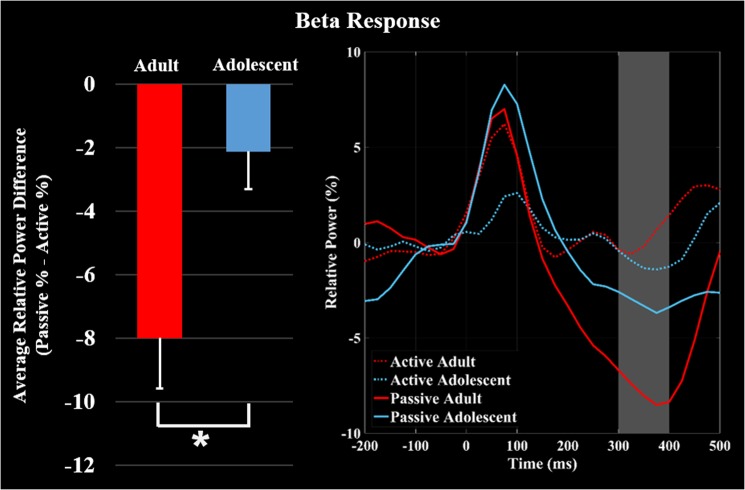
Average of the neural time series extracted from the peak voxel in the beta grand averaged beamformer images for the adolescents (blue) and adults (red). The solid lines represent the neural time courses for the passive condition, while the dashed lines represent the neural time courses for the active condition. The bar graph shows the amount of attenuation (Passive – Active) of the average relative power of beta event related desynchronization (ERD) during the 300–400 ms time window. Significant differences in the magnitude of attenuation are denoted by the asterisk (P ≤ 0.05). As shown, the adults had greater attenuation (e.g., gating) of the beta ERD relative to the adolescents during the isometric ankle plantarflexion task.

The beta (18–26 Hz) ERD was imaged from 300 to 400 ms using passive and active trials and a baseline period of −125 to −25 ms. The beta ERD was also centered on the leg region of the contralateral somatosensory cortex (Fig. 2C). Virtual sensor time series were extracted from the peak of the response separately for the active and passive conditional images per participant, and then averaged across the 300 to 400 ms time window. The power of the somatosensory ERD response was significantly weaker during the active condition (*P* < 0.001, Fig. 2C). Additionally, the the attenuation of the beta ERD in the active condition was greater in the adults (*P* = 0.029, Fig. 4).

### Correlational Results

The magnitude of attenuation for the alpha-beta and gamma ERS and beta ERD were not related to subject age (*P*s > 0.05).

## Discussion

This investigation used MEG and advanced beamforming to quantify changes in the somatosensory cortical oscillations while sitting quietly (e.g., passive condition) and while producing an ankle plantarflexion isometric force. The data-directed methodology utilized in this investigation revealed that for both conditions there were an alpha-beta (8–30 Hz, 0–125 ms) and a gamma (38–80 Hz, 0–100 ms) ERS in the contralateral somatosensory cortices near the leg region that occurred immediately after the peripheral stimulation. These oscillatory changes were followed by a beta ERD (18–26 Hz) that occurred in the later time window (300–400 ms). When compared with the passive condition, all of these frequency specific cortical oscillations were attenuated when participants produced the isometric force (i.e., during the active condition). Furthermore, the adolescents demonstrated greater attenuation of the alpha-beta ERS, while the adults had greater attenuation of the beta ERD. These results imply that altered attenuation of the respective cortical oscillations might be central to the altered or under-developed somatosensory processing and motor performance characteristics previously reported in the behavioral literature on adolescents^8,9,13–16^.

The strength of the gamma ERS in the somatosensory cortex was significantly weaker during the active condition, but the amount of attenuation was not different between the adults and adolescents. This may imply that this frequency specific somatosensory oscillation is mature by adolescents and thus would not likely underlie the motor control differences previously reported for adolescents. Gamma cortical oscillations are typically associated with higher-order information processes, such as attention^26–28^. Prior MEG research has shown that the gamma ERS in the somatosensory cortex tends to be stronger when the participants attend to the peripheral stimulation^29^. Based on this evidence, it is possible that the reduction in the gamma ERS seen during the motor task may be driven by allocation of attentional resources. In other words, the somatosensory gamma ERS was gated during the movement because more attentional resources were allocated towards generating the isometric muscular force.

These results also showed that the strength of the alpha-beta ERS in the somatosensory cortex was significantly weaker while the participants generated the isometric ankle plantarflexion force. This conditional effect is aligned with the prior results from EEG with humans and animal model studies^17–20,30–32^. Additionally, our analysis identified that the adolescents exhibit a greater attenuation of the alpha-beta ERS while generating the isometric force. This may indicate that the adolescents have greater difficulty processing somatosensory feedback during volitional motor actions. Similar to the conjecture put forth in the preceding paragraph, we suspect that the excessive hyper-gating may be a result of allocation of resources that are essential for simultaneously processing the sensory feedback and generating an isometric force. This gating during a motor action may stop necessary sensory information from reaching higher cortical levels, thus contributing to immature motor patterns characteristic of adolescents. Alternatively, it could be that the circuitry in local neural populations has yet to be fully optimized and the combined sensorimotor input results in a strong suppression of certain activity.

In contrast to the alpha-beta ERS, the somatosensory beta ERD occurred in a later time well after the stimulation. This response is considered to be a rebound or resetting of the somatosensory cortical oscillations^33,34^. Hence, it is possible that the adolescents uncharacteristically reset the somatosensory cortical oscillations while generating the isometric force, while the adults tend to continue to process the ongoing somatosensations. Alternatively, it has been postulated that this later oscillatory activity may be a result of the sensory information generated through electrically stimulating the Ia afferents that interface with the muscle spindles and/or peripheral alpha motor neurons^25^. This is based on the premise that the Hoffman reflex can be modulated by stimulation of the Ia afferents with a submaximal electrical stimulus^35–37^. A muscle twitch is produced by this reflexive pathway through the monosynaptic connections linking the Ia afferents and alpha motor neurons in the anterior horn of the spinal cord. Throughout adolescence, the magnitude of the Hoffman reflex scales with age^35^. Therefore, it is plausible that the altered beta ERD might be linked with the maturation of the Hoffman reflex, but further studies are needed.

Our results showed that all of the frequency specific somatosensory cortical oscillations were reduced during the production of an ankle plantarflexion isometric force. Further, attenuation of the alpha-beta somatosensory ERS during isometric force production appears to be greater in adolescents relative to adults. In contrast, adults have a greater attenuation of the beta ERD. These results imply that alterations of frequency specific somatosensory cortical oscillations may partly underlie the altered motor performance characteristics seen during adolescence.

## Material and Methods

### Subjects

Nineteen adolescents (Age = 14.8 ± 2.5 yrs.; Female = 9) and nineteen adults (Age = 36.8 ± 5.0; Female = 9) with no neurological or musculoskeletal impairments were enrolled in this study. All participants were right-handed. The Institutional Review Board at the University of Nebraska Medical Center reviewed and approved the protocol for this investigation, and all participants or their legal guardians provided informed consent or assent prior to participation in the study. All methods were carried out in accordance with relevant guidelines and regulations.

### MEG Data acquisition and experimental paradigm

All MEG recordings were conducted in an ETS-Lindgren one-layer magnetically shielded room (Eura, Finland) with active shielding engaged for advanced environmental noise compensation. During data acquisition, participants were monitored via real-time audio-video feeds from inside the shielded room. Neuromagnetic responses were acquired with a bandwidth of 0.1–330 Hz and were sampled continuously at 1 kHz using an Elekta MEG system (Helsinki, Finland) with 306 sensors, including 204 planar gradiometers and 102 magnetometers. With the use of the MaxFilter software (Elekta), each MEG dataset was individually corrected for head motion and subjected to noise reduction using the signal space separation method with a temporal extension^38^.

The participants were seated in a nonmagnetic chair with their head positioned within the MEG helmet-shaped sensor array. Unilateral electrical stimulation was applied to the right posterior tibial nerve using external cutaneous stimulators that were connected to a Digitimer DS7A constant-current stimulator system (HW Medical Products, Neuberg, Germany). During stimulation, each participant sat quietly focused on a fixation cross (passive condition), or performed an ankle isometric force target matching task (active condition). During both the passive and active conditions, single 0.2 ms constant-current square waves were presented using an interstimulus interval that randomly varied between 1800 and 2200 ms. The amplitude of the pulses were set to the threshold required to elicit a visible flexor twitch in the big toe and was constant for both conditions.

**Figure 5:**
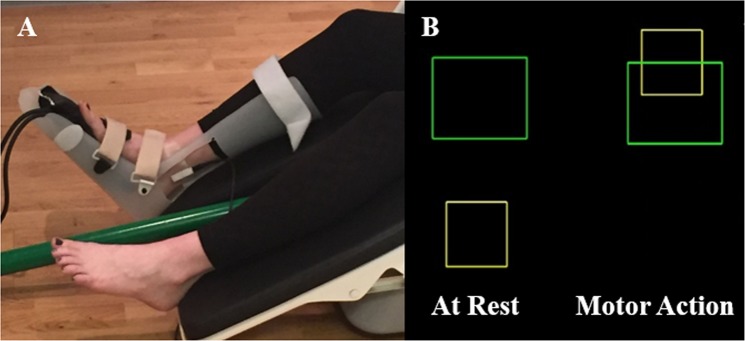
(**A**) Participant seated in the MEG chair with the electrical stimulator placed over the tibial nerve and the custom pneumatic ankle force system on their right leg. (**B**) Exemplary visual feedback displayed to the participant. The isometric ankle plantarflexion forces generated by the participant animated the vertical position of a yellow box’s position on the screen. The goal of the task was to generate an isometric force that shot the yellow box through the presented green target box.

During the active condition, the participants were instructed to generate an isometric ankle plantarflexion force with the right leg. A custom-built magnetically-silent pneumatic force transducer was used to measure the isometric forces and was concurrently sampled at 1 kHz along with the MEG data (Fig. 5^39,40^;). The experimental task consisted of the participant generating an isometric force that would animate a box to ascend vertically and shoot through a target box. The target boxes had vertical positions that were between 5–30% of the participant’s maximum isometric ankle plantarflexion force and their positions were randomly determined. The respective boxes were visually displayed on a back-projection screen that was ~1 meter in front of the participant at eye level. Each participant generated ~200 isometric plantarflexion forces. Each trial lasted 1500 ms and was followed by an 800 ms rest period. Only those trials where electrical stimulation occurred during the isometric force were selected for analysis.

### MEG Coregistration

Four coils were affixed to the head of the participant and were used for continuous head localization during the MEG experiment. Prior to the experiment, the location of these coils, three fiducial points, and the scalp surface were digitized to determine their three-dimensional position (Fastrak 3SF0002, Polhemus Navigator Sciences, Colchester, VT, USA). Once the participant was positioned for the MEG recording, an electric current with a unique frequency label (*e.g*., 322 Hz) was fed to each of the four coils. This induced a measurable magnetic field and allowed for each coil to be localized in reference to the sensors throughout the recording session. Since the coil locations were also known in head coordinates, all MEG measurements could be transformed into a common coordinate system. With this coordinate system (including the scalp surface points), each participant’s MEG data were coregistered with native space neuroanatomical MRI data using three external landmarks (*i.e*., fiducials) and the digitized scalp surface points prior to source space analyses. The neuroanatomical MRI data were aligned parallel to the anterior and posterior commissures and transformed into standardized space following source imaging using BESA MRI (Version 2.0; BESA GmbH, Gräfelfing, Germany).

### MEG Pre-processing, time-frequency transformation, & statistics

Artifact rejection was based on a fixed threshold method, supplemented with visual inspection. The number of trials were balanced between age group and condition, and were tested for differences using a mixed model ANOVA (Adolescent/Adult Group X Active/Passive Condition), which showed no significant difference between the number of trials per age group or condition (*Ps* > 0.05). The continuous magnetic time series was divided into epochs of 1100 ms in duration (−500 to 600 ms), with the onset of the electrical simulation defined as 0 ms and the baseline defined as −200 to 0 ms. Artifact-free epochs for each sensor were transformed into the time-frequency domain using complex demodulation and averaged over the respective trials. These sensor-level data were normalized per time-frequency bin by using the respective bin’s baseline power, which was calculated as the mean power during the baseline (−200 to 0 ms). The specific time-frequency windows used for imaging were determined by statistical analysis of the sensor-level spectrograms across the entire array of gradiometers. Briefly, each data point in the spectrogram was initially evaluated using a mass univariate approach based on the general linear model. To reduce the risk of false positive results while maintaining reasonable sensitivity, a two-stage procedure was followed to control for Type 1 error. In the first stage, one-sample t-tests were conducted on each data point and the output spectrogram of t-values was thresholded at p < 0.05 to define time-frequency bins containing potentially significant oscillatory deviations across all participants and conditions. In stage two, time-frequency bins that survived the threshold were clustered with temporally and/or spectrally neighboring bins that were also above the (*p* < 0.05) threshold, and a cluster value was derived by summing all of the t-values of all data points in the cluster. Nonparametric permutation testing was then used to derive a distribution of cluster-values and the significance level of the observed clusters (from stage one) were tested directly using this distribution^41,42^. For each comparison, at least 10,000 permutations were computed to build a distribution of cluster values.

### MEG Source imaging

A minimum variance vector beamforming algorithm was employed to calculate the source power across the entire brain volume using a spherical head model^43^. The single images were derived from the cross spectral densities of all combinations of MEG sensors and the solution of the forward problem for each location on a grid specified by input voxel space. Following convention, the source power in these images was normalized per subject using a separately averaged pre-stimulus noise period of equal duration and bandwidth^44–46^. Thus, the normalized power per voxel was computed over the entire brain volume per participant at 4.0 × 4.0 × 4.0 mm resolution. Each participant’s functional images, which were co-registered to anatomical images prior to beamforming, were transformed into standardized space using the transform previously applied to the structural MRI volume and spatially resampled. MEG pre-processing and imaging used the Brain Electrical Source Analysis (BESA) software (BESA v6.0; Grafelfing, Germany).

Time series analysis was subsequently performed on the neural activity extracted from the peak voxel in the grand-averaged beamformer images (see Results below). The virtual neural time courses were created by applying the sensor weighting matrix derived through the forward computation to the preprocessed signal vector, which resulted in a time series with the same temporal resolution as the original MEG recording^47–49^. Once the neural time courses were extracted, they were transformed into the time-frequency domain, and the two orientations for each peak voxel per individual were combined using a vector-summing algorithm. The power of these time courses, relative to baseline, was averaged across the window of interest for each individual to assess the key oscillatory responses. The data were then collapsed across groups and paired-samples t-tests were used to test if condition had an effect on the power of the somatosensory responses. Further, to test if the attenuation of the somatosensory response differed between groups, the average difference of the power (Passive – Active) during time-frequency bins of interest were tested using a two-sample t-test. Pearson’s correlations were ran to assess the relationship between the magnitude of attenuation of the individual signals and the subject age.

It should be noted that the methodology described in the proceeding sections were similar to what has been employed in our prior experimental work^21,24,25,39,40,50–55^.

## Data Availability

The de-identified datasets generated during and/or analyzed during the current study are available from the corresponding author on reasonable request and IRB approval.
